# Dynamics of HIV PrEP use and coverage during and after COVID-19 in Germany

**DOI:** 10.1186/s12889-024-19198-y

**Published:** 2024-06-25

**Authors:** Daniel Schmidt, Yannick Duport, Christian Kollan, Ulrich Marcus, Sara Iannuzzi, Max von Kleist

**Affiliations:** 1 Department of Infectious Disease Epidemiology, Robert-Koch Institute, Berlin, Germany; 2 Project Groups, Robert-Koch Institute, Berlin, Germany; 3 Department of Mathematics and Computer Science, Freie Universität Berlin, Berlin, Germany

**Keywords:** HIV-PrEP users, COVID-19, Germany, PrEP need, PrEP coverage, Pharmaceutical prescription data

## Abstract

**Background:**

Pre-exposure prophylaxis (PrEP) with oral emtricitabine/tenofovir disoproxil (FTC/TDF) proved highly efficient in preventing HIV. Since 09/2019, FTC/TDF-PrEP is covered by health insurances in Germany, if prescribed by licensed specialists. However, methods to longitudinally monitor progress in PrEP implementation in Germany are lacking.

**Methods:**

Utilizing anonymous FTC/TDF prescription data from 2017-2021, we developed a mathematical model to disentangle HIV-treatment from PrEP prescriptions, as well as to translate PrEP prescriptions into number of PrEP users. We used the model to estimate past- and future PrEP uptake dynamics, to predict coverage of PrEP needs and to quantify the impact of COVID-19 on PrEP uptake on a national and regional level.

**Results:**

We identified significant (*p*<0.01) decelerating effects of the first- and second COVID-19-lockdown on PrEP uptake in 04/2020 and 12/2020. We estimated 26,159 (CI: 25,751-26,571) PrEP users by 12/2021, corresponding to 33% PrEP coverage of people in need. We projected 64,794 (CI: 62,956-66,557) PrEP users by 12/2030, corresponding to 81% PrEP coverage. We identified profound regional differences, with high PrEP coverage and uptake in metropoles and low coverage in more rural regions.

**Conclusions:**

Our approach presents a comprehensive solution to monitor and forecast PrEP implementation from anonymous data and highlighted that the COVID-19 pandemic significantly decelerated PrEP uptake in Germany. Moreover, slow PrEP uptake in rural areas indicate that structural barriers in PrEP care, education or information exist that may hamper the goal of ending the AIDS epidemic by 2030.

**Supplementary Information:**

The online version contains supplementary material available at 10.1186/s12889-024-19198-y.

## Background

Human immunodeficiency Virus (HIV-1) infection constitutes one of the most severe pandemics to date, with 2–3 infections per minutes, globally [1]. While HIV can be treated with effective antiretroviral treatment (ART) to prevent acquired immunodeficiency syndrome (AIDS) and death [2] as well as HIV transmission, currently no cure is available [3] and neither an effective vaccine [4]. HIV can be efficiently prevented by condom usage, HIV treatment as prevention, as well as needle exchange for people who inject drugs. In addition to these prevention tools, pre-exposure prophylaxis (PrEP) with oral emtricitabine/tenofovir disoproxil fumarate (FTC/TDF) is nowadays perceived as a highly efficient tool when taken daily [5]. In men-who-have-sex with men (MSM) FTC/TDF-PrEP may even be taken on-demand [6, 7], while in heterosexual cis-gender women on-demand regimen are being discussed [8].

About 90,800 individuals in Germany were HIV infected in 2021, of which the majority (61%) are MSM [9]. While incidences are decreasing since 2016, an estimated 1800 new HIV infections occurred in 2021, of which approximately 1000 (56%) were in MSM [9]. To further prevent HIV infection, HIV-PrEP with daily FTC/TDF is covered by German statutory health insurances (SHI) for persons with high risk of HIV infection since September 2019 [10, 11], if prescribed by certified HIV specialists or physicians who received specialized training.

Unlike other European countries [12], the German health system is highly decentralized, without electronic patient records, to date. Hence, there is no systematic recording of the number of PrEP users in different parts of the country, implying major difficulties in monitoring PrEP usage, roll-out & HIV prevention goals, and in identifying regional barriers to PrEP use in Germany. In the absence of an electronic patient recording system, PrEP prescription data may be used, as e.g. demonstrated in earlier analyses of PrEP use in the US [13–17]. Electronic prescription data is available for research under certain regulations representing all individuals with statutory health insurance in Germany, which amounts to about 74 million individuals [18]. The introduction of PrEP as SHI benefit in Germany has been scientifically evaluated and a national PrEP surveillance is currently being established at the Robert Koch Institute [19–21]. Estimates from these projects calculated between 15,600–21,600 PrEP users in Germany as of June 2020 [22] and approximately 32,000 PrEP users by the end of 2022 [20, 23]. However, these numbers are point estimates. Trends and effects due to changes in supply or behavior, such as the effects of COVID-19, cannot be evaluated and predictions are not possible based on previous analysis. Therefore, a method to reliably model past and future PrEP use and coverage for people *in need* of PrEP is still lacking.

The goal of our project was therefore to utilize FTC/TDF prescription data to estimate PrEP use and coverage in Germany. By modelling the data, we extract the current and future status, temporal dynamics and regional differences in PrEP uptake, as well as the impact of COVID-19 on PrEP uptake in Germany.

## Methods

### Data source

Health insurance is compulsory in Germany, with almost 90% of German residents covered by statutory health insurance [18]. PrEP with FTC/TDF can be prescribed via statutory health insurance by certified HIV specialists, whereas other physicians need to undergo training or can prescribe PrEP on a self-payer basis [11]. Statutory health insurance takes a central role in PrEP service delivery in Germany since 89.5% of PrEP users at HIV specialists receive PrEP through statutory health insurance [24]. Statutory health insurance reimburses pharmacies for dispensed prescribed drugs via specialized pharmacy billing centers, which generate spatially resolved, electronically recorded prescription details.

ART prescription data were provided by Insight Health™ and analyzed for the years 2017–2021. The data were collected on a monthly basis from billing centers that processed all reimbursed prescriptions from pharmacies based on the date of redemption at the counter. Regional assignment of prescription data to federal states is the operating site of the prescribing physician. The provider claimed a coverage of > 99% within the SHI prescription market. The recorded numbers of prescribed standard units (i.e., numbers of tablets) sold of single tablet FTC/TDF were used for this study as FTC/TDF is the only drug with approval for PrEP in Germany.

The data include all single FTC/TDF, regardless of whether they were used as part of HIV treatment, or short-term post-exposure prophylaxis (PEP) or are given as FTC/TDF for PrEP. Triple substance single tablet regimen containing FTC and TDF can be distinguished and are not included in the dataset. The data is anonymized with no individual information and no treatment indication available. Further, no accessible national data source for the SHI system currently exists, which would allow the validation of prescriptions according to treatment indication.

The recording and use of these data are regulated by the social security law (§300 SGB V), no ethical approval and informed consent were required since this routinely collected, anonymized secondary data cannot be traced back to individual patients.

### Estimating PrEP needs

PrEP needs were estimated based on EMIS-2017 data [22] with a slightly modified calculation. PrEP need was defined as the combination of “subjective need” (= intention to use PrEP) and “objective need” (= at least two non-steady condomless anal intercourse partners reported for the last 12 months). EMIS data were stratified by federal state and extrapolated to the estimated total population of gay men after adjustment for a likely survey participation bias.

### Generation of a continuous trajectory from prescription data

**Fig. 1:**
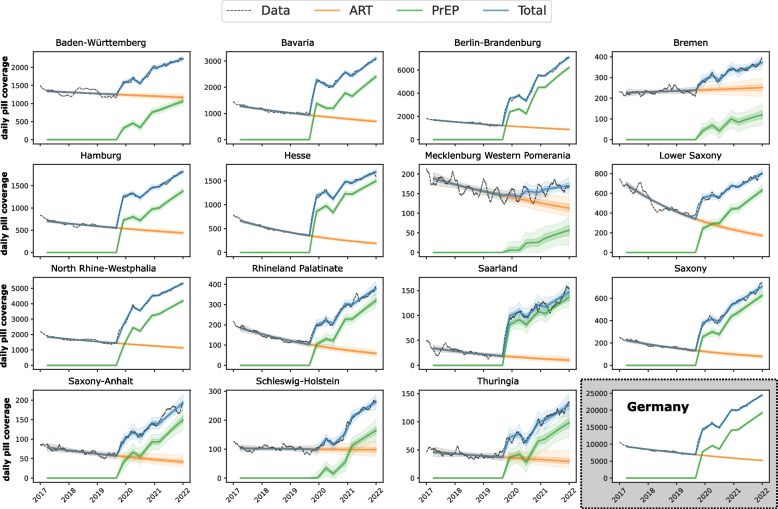
Prescription data for each German federal state as well as the entire country. Daily pill coverage from prescription data is highlighted with a black dashed line, whereas model predictions for the number of ART-PEP prescriptions, PrEP prescriptions and their sum are highlighted in orange, green and blue. Dark and light shading denotes interquartile ranges and 95% confidence intervals

Our data set contained the number of FTC/TDF prescriptions per month for the different package sizes available in Germany. Package sizes of 28, 30 and 35 tablets were defined as one-month prescription, package sizes of 84 and 90 tablets as three-month prescription. For each prescription we drew a random date within the month it was prescribed and incremented the next *k* days by one, where *k* denotes the prescribed package size. Using this procedure, we obtain a trajectory of daily FTC/TDF pill coverage, Fig. 1.

### Mathematical model

FTC/TDF is used for HIV treatment, PEP, as well as PrEP. However, since PrEP is covered by SHI as of September 2019 with FTC/TDF remaining the only approved PrEP regimen in Germany, FTC/TDF prescription numbers have increased significantly after September 2019. As part of this modelling exercise we aim to distinguish between the use of FTC/TDF for PrEP vs. HIV treatment and PEP. For this purpose, we developed a simple ordinary differential equation model capable of predicting the daily FTC/TDF pill coverage for PrEP vs. other uses, at both the federal and state levels. Our model consists of two variables ${Y}_{ART}$ and ${Y}_{PrEP}$ that model the daily FTC/TDF pill coverage for HIV therapy (and PEP) vs. PrEP prescriptions:

1 $$\frac d{dt}Y_{ART}\left(t\right)=k_{ART}\bullet Y_{ART}\left(t\right)\;(\mathrm{prescriptions}\;\mathrm{for}\;\mathrm{ART}\;\mathrm{and}\;\mathrm{PEP})$$

2 $$\frac d{dt}Y_{PrEP}\left(t\right)=k_{PrEP}\left(t\right)\bullet\left(N_{iN}-c_{oD}\bullet c_{SHI}\bullet Y_{PrEP}\left(t\right)\right)\;(\mathrm{prescriptions}\;\mathrm{for}\;\mathrm{PrEP})$$

3 $${Y_{Tot}\left(t\right)=Y}_{PrEP}\left(t\right)+Y_{ART}\left(t\right),\;(\mathrm{total}\;\mathrm{prescriptions})$$

where ${c}_{oD}\bullet {c}_{SHI}$ is a constant that translates daily PrEP pill coverage into PrEP users (outlined below). For${Y}_{ART}\left(t\right)$, we assume linear kinetics with rate${k}_{ART}$, reflecting the dynamics of FTC/TDF use in antiretroviral therapy HIV therapy and PEP. In the case of PrEP prescriptions ${Y}_{PrEP}\left(t\right)$ we assume that there were none in the data source, which represents SHI reimbursed prescriptions, before PrEP coverage by SHI (before Sept/2019), and that prescriptions tend to increase over time and may eventually saturate when the number of people in need of PrEP ${N}_{iN}$ is reached (22). In the model, the rate of PrEP uptake ${k}_{PrEP}(t)$ changes between distinct episodes that model COVID-19 effects on PrEP, Table 1 below. In total, Germany experienced two major COVID-19 lock-downs (Apr.-Jun. ‘20 and Dec.’20-Feb. ‘21). In total, we modelled six PrEP episodes, which are, in addition to the lock-down, characterized by an initially rapid uptake of SHI covered PrEP, probably by those in anticipation of this prevention tool.

**Table 1:** Different episodes of PrEP uptake considered in the model

Episode	Description
Sep. 1st ‘19—Nov. 30th ‘19	Switching from self-paid PrEP to SHI-reimbursed PrEP and initial “run” on PrEP for those in anticipation
Dec. 1St ‘19—Mar. 31st ‘20	Before first COVID-19 lock-down
Apr. 1st ‘20—Jun. 30th ‘20	First COVID-19 lock-down
Jul. 1St ‘20—Nov. 30th ‘20	Before second lock-down
Dec. 1st ‘20—Feb. 28th ‘21	Second COVID-19 lock-down
Mar. 1st ‚21—Dec. 31st 21	After second lockdown

We assumed that each lock-down, as well as the initial phase of PrEP affected the rate of daily PrEP prescriptions for three consecutive months, which is motivated by the most frequently used package sizes (90 tablets).

### Translating statutory health insurance prescriptions into number of daily and on-demand PrEP users

For daily oral PrEP, the number of prescribed tablets would theoretically equal the number of person-days on PrEP. However, in a recent study, Schmidt et al. reported that the number of days on PrEP was 0.91 for daily users (hence, each pill covers 1/0.91 = 1.1 days on average). Furthermore, 18.9% of PrEP users, take it on-demand [25]. On-demand users, took PrEP 58% of the time, hence each pill would last for 1.72 days on average. Consequently, we can convert the number of prescribed pills through statutory health insurances to the number of PrEP users ${c}_{oD}= \left(\frac{0.189}{0.58}+\frac{0.811}{0.91}\right)=1.22$. Lastly, while 89.5% of all PrEP users are SHI covered, the total number of prescriptions should be corrected for not statutory health insured individuals ${c}_{SHI}=\frac{1}{0.895}=1.12$ [24, 26].

### Model fitting

To obtain model parameters and initial values, the model was fitted to the number of daily FTC/TDF prescriptions ${Y}_{data}\left(t\right)$, by minimizing the residual sum of squares (RSS):

4 $$\underset{\theta }{\text{min}}{\Vert {{Y}_{data}\left(t\right)-Y}_{Tot}\left(t,\theta \right)\Vert }_{2}^{2}$$

where $\theta = \left\{{k}_{ART}, {k}_{PrEP}\left({t}_{1}\right),{\dots ,{k}_{PrEP}\left({t}_{6}\right), Y}_{ART}\left({t}_{0}\right),{Y}_{PrEP}\left({t}_{0}\right)\right\}$ denote the model parameters (rate parameters and intitial conditions). Parameters were determined for the individual German federal states, as well as for the entire country. Parameter optimization was done in two steps: First, the model was fitted against all datapoints before Sept./2019 to determine the initial value ${Y}_{ART}\left({t}_{0}\right)$ and the rate constant ${k}_{ART}$. Subsequently, the remaining rate constants ${k}_{PrEP}\left({t}_{1}\right), \dots , {k}_{PrEP}\left({t}_{6}\right)$ were determined by fitting the model against all datapoints, as described above.

### Uncertainty estimation

To estimate uncertainty in the data, model parameters and model predictions, we performed a parametric re-sampling technique in two steps: First, the total number of FTC/TDF prescriptions per month ${\widehat{Y}}_{data}\left(t\right)$ was sampled from a binomial distribution:

5 $${\widehat{Y}}_{data}\left(t\right)\sim \mathcal{B}\left({N}_{need}\left(t\right),{p}_{data}\left(t\right)\right)$$

where ${N}_{need}\left(t\right)$= ${N}_{iN}+ {Y}_{ART}\left(t\right)$ denotes the total number of people needing FTC/TDF, either for PrEP ${N}_{iN}$, or for HIV therapy (and PEP) ${Y}_{ART}.$ For the latter, we used the model-simulated ${Y}_{ART}\left(t\right)$ as outlined above. The parameter ${p}_{data}\left(t\right)= \left({N}_{30}\left(t\right)+ {N}_{90}\left(t\right)\right)/{N}_{need}\left(t\right)$ denotes the probability of FTC/TDF prescription, where ${N}_{30}\left(t\right)$ and ${N}_{90}\left(t\right)$ denote the number of one-month (28, 30 and 35 tablets) and three-month (84 and 90 tablets) prescriptions at time t in the dataset. In a second step, the number of one-month (N_30_) vs. three-month (N_90_) prescriptions were sampled from a binomial distribution:

6 $${\widehat{N}}_{30}\left(t\right)\sim \mathcal{B}\left({\widehat{Y}}_{data}\left(t\right),{p}_{30}\left(t\right)\right)$$

7 $${\widehat{N}}_{90}\left(t\right)={\widehat{Y}}_{data}\left(t\right)-{\widehat{N}}_{30}\left(t\right)$$

where ${p}_{30}\left(t\right)= {N}_{30}\left(t\right)/\left({N}_{30}\left(t\right)+ {N}_{90}\left(t\right)\right)$ is the probability of a one-month prescription.

## Results

### Mathematical model can distinguish between PrEP and ART prescriptions.

Using the developed mathematical model (*Methods* section) we were able to distinguish between FTC/TDF prescriptions used for PrEP vs. ART (+ PEP), as shown in Fig. 1 for each federal sate in Germany. Between 2017 and September 2019, i.e. before PrEP became available via SHI, the total number of persons using FTC/TDF for ART (+ PEP) steadily decreased from 10,000 to about 7,000 in Germany (Fig. 1, lower right panel). The decreasing trend of FTC/TDF prescriptions prior to Sept/2019 was evident in all 16 German federal states except Bremen, which denotes low prescription numbers and the smallest state (in terms of size and population) in Germany. The decreasing trend of ART prescriptions was followed by a sharp increase coinciding with the introduction of SHI-PrEP in September 2019 that was evident in all, but a few population-wise smaller states (Mecklenburg Western Pomerania, Schleswig–Holstein, Bremen). According to our model, this sharp increase was solely attributable to PrEP prescriptions. Following this initial increase in PrEP uptake, we observed a further increase in FTC/TDF prescriptions over the observation time horizon (until Dec. 2021). However, two time points of decreasing FTC/TDF prescriptions became apparent in April 2020, as well as in December 2020, as visible when considering the entire German data set (Fig. 1, lower right panel). However, we observed differences between distinct German federal states, which could be attributable to differences in uptake, or of statistical nature (small sample sizes).

### Effect of COVID-19 lockdowns on PrEP prescription dynamics

Next, we evaluated the effect of COVID-19 lockdowns on the uptake of PrEP. Using our model, we could quantify whether the model-predicted rate of PrEP uptake would be different before and after the two lockdowns (before April 2020 vs. after; before December 2020 vs. after). When considering the entire data set (all of Germany), we observed a significant (p < 0.01) decelerating effect of the first- and second lockdown on the rate of PrEP uptake, Supplementary Fig. S1-2. When analyzing COVID-19 effects in individual German federal states we either observed significant decreases in PrEP uptake, or statistically inconclusive changes due to small sample sizes.

### Current and projected PrEP coverage and regional differences

With our model we were able to calculate the absolute number of PrEP users in Germany and in each federal state. We estimated that the number of PrEP users in Germany was 19,260 (CI: 18,923–19,572) by the end of 2020 and 26,159 (CI: 25,751–26,571) by the end of 2021, Table 2. Notably, the majority of PrEP users are situated in Berlin-Brandenburg and North Rhine-Westphalia, both known for their large MSM communities [22, 27]. Considerably less PrEP users were allocated to eastern German federal states and rural territorial states. Using the model, we could project these numbers into the future, estimating that if the current dynamics of PrEP uptake remain, then there will be 49,308 (CI: 47,627–51,056) PrEP users in Germany by the end of 2025 and 64,794 (CI: 62,956–66,557) by the end of 2030.

**Table 2:** Estimated PrEP users by federal state (Median absolute number [95% CI]) over time

federal state	2019–12	2020–06	2020–12	2021–06	2021–12	2025–12	2030–12
Baden-Württemberg	**480** [381, 587]	**453** [325, 585]	**1060** [934, 1178]	**1247** [1122, 1366]	**1449** [1290, 1612]	**2844** [2061, 3511]	**4137** [2860, 5082]
Bavaria	**1819** [1714, 1932]	**1622** [1480, 1756]	**2368** [2245, 2485]	**2676** [2562, 2780]	**3274** [3119, 3428]	**6703** [6134, 7227]	**8868** [8279, 9339]
Berlin-Brandenburg	**3336** [3215, 3465]	**3047** [2880, 3215]	**6125** [5990, 6277]	**7119** [7006, 7235]	**8464** [8270, 8637]	**14,669** [14220, 15070]	**17,143** [16864, 17357]
Bremen	**68** [24, 110]	**59** [5, 113]	**128** [76, 181]	**135** [78, 189]	**163** [91, 236]	**360** [2, 574]	**527** [0, 744]
Hamburg	**1012** [932, 1092]	**977** [874, 1078]	**1337** [1246, 1417]	**1577** [1498, 1657]	**1875** [1761, 1989]	**3253** [2951, 3485]	**3813** [3598, 3927]
Hesse	**1209** [1140, 1277]	**1139** [1047, 1237]	**1670** [1587, 1745]	**1814** [1754, 1879]	**2036** [1940, 2130]	**3432** [2878, 3847]	**4496** [3733, 4954]
Mecklenburg Western Pomerania	**8** [0, 37]	**32** [0, 73]	**40** [0, 80]	**61**[24, 96]	**77** [24, 124]	**191** [0, 397]	**312** [0, 615]
Lower Saxony	**345** [293, 402]	**403** [337, 470]	**602** [547, 661]	**710** [665, 757]	**859** [786, 931]	**1858** [1397, 2234]	**2744** [2035, 3253]
North Rhine-Westphalia	**2116** [1993, 2239]	**3025** [2854, 3201]	**4442** [4295, 4585]	**5002** [4865, 5135]	**5692** [5505, 5893]	**9848** [9072, 10650]	**12,837** [11896, 13676]
Rhineland Palatinate	**149** [115, 183]	**166** [123, 209]	**309** [274, 344]	**361** [329, 389]	**436** [386, 487]	**946** [692, 1172]	**1410** [998, 1716]
Saarland	**114** [95, 135]	**111** [86, 137]	**145** [124, 168]	**160** [143, 177]	**186** [159, 215]	**366** [164, 517]	**530** [168, 721]
Saxony	**362** [316, 402]	**377** [321, 434]	**612** [560, 663]	**728** [688, 770]	**849** [785, 915]	**1637** [1259, 1937]	**2269** [1709, 2618]
Saxony-Anhalt	**64** [40, 88]	**75** [46, 106]	**126** [99, 153]	**158** [133, 180]	**203** [169, 241]	**500** [341, 638]	**739** [504, 895]
Schleswig–Holstein	**8** [0, 34]	**21** [0, 57]	**102** [62, 139]	**181** [144, 216]	**224** [172, 274]	**529** [255, 768]	**843** [339, 1197]
Thuringia	**52** [32, 71]	**39** [15, 63]	**91** [65, 116]	**111** [85, 132]	**134** [99, 167]	**304** [114, 461]	**471** [111, 698]
**Germany**	**11,199** [10920, 11471]	**11,647** [11298, 11984]	**19,261** [18923, 19572]	**22,204** [21933, 22501]	**26,159** [25751, 26571]	**49,308** [47627, 51056]	**64,794** [62956, 66557]

**Fig. 2:**
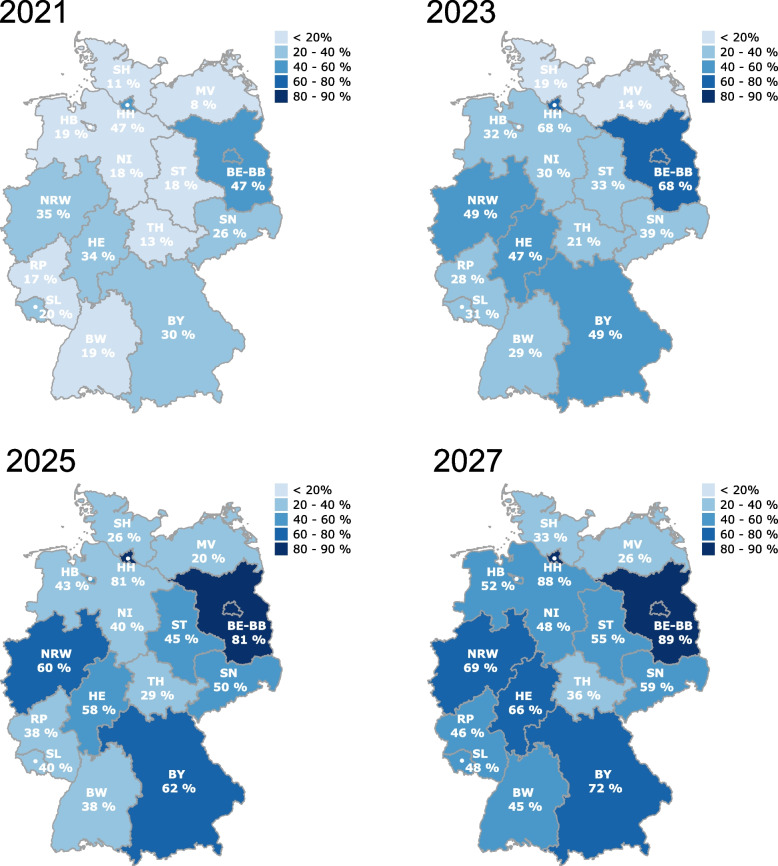
Calculated and projected PrEP coverage for each German federal state. PrEP coverage was computed as the fraction of PrEP users among individuals in need ${\text{N}}_{\text{iN}}$. Prescription data was available until the end of 2021 (upper left panel), whereas the other panels denote model predictions. BW = Baden-Württemberg, BY = Bavaria, BE-BB: Berlin-Brandenburg; HB = Bremen; HH = Hamburg; HE = Hesse; MV = Mecklenburg Western Pomerania; NI = Lower Saxony; NRW = North Rhine-Westphalia; RP = Rhineland Palatinate; SL = Saarland; SN = Saxony; ST = Saxony-Anhalt; SH = Schleswig–Holstein; TH = Thuringia

Next, we estimated which proportion of individuals ‘in need’ [22] received PrEP in the past (denoted as ‘coverage’) and we projected, assuming that PrEP uptake dynamics remained, which proportion will be covered in the future, Fig. 2, Supplementary Table S1. Using our model, we estimated that PrEP coverage in Germany was 24% (CI: 24–25%) by the end of 2020 and 33% (CI: 32–33%) by the end of 2021 (Supplementary Table S1). We identified profound differences between different federal states, with high PrEP coverage above the German average in Berlin-Brandenburg, Hamburg, North Rhine-Westphalia and Hesse and less coverage in most eastern German federal states and more territorial states, Fig. 2. Projecting PrEP coverage into the future, we found that 62% (CI: 59–64%) coverage would be achieved by the end of 2025 and 81% (CI: 79–83%) by the end of 2030 (Supplementary Table S1).

We then projected when 25, 50, 75 and 90% PrEP coverage would be achieved in Germany and in the individual federal states, Table 3. According to our predictions, these goals have been/would be achieved in Germany in 21/03 (CI: 21/03–21/04), 24/02 (CI: 23/11–24/05), 29/01 (CI: 28/05–29/10) and 35/08 (CI: 34/05–36/11). Again, we observe large differences between distinct federal states with PrEP 50% coverage goals already achieved in 2022 in the major German cities (Berlin, Hamburg) and projected to be achieved in 2024 in North Rhine-Westphalia, Hesse and Bavaria, whereas Mecklenburg-Western Pomerania and Schleswig Holstein would be the last states to achieve the 50% coverage goal.

**Table 3:** Model predicted median date to reach 10, 25, 50, 75 and 90% PrEP coverage of individuals in need [95% CI] (mm/YY [mm/YY])

state	10% Coverage	25% Coverage	50% Coverage	75% Coverage	90% Coverage
Baden-Württemberg	**09/2020** [04/20, 10/20]	**03/2023** [07/22, 01/25]	**06/2029** [09/26, 01/38]	**03/2040** [11/33, ≥ 2050]	** ≥ 2050** [04/43, ≥ 2050]
Bavaria	**10/2019** [10/19, 11/19]	**07/2021** [06/21, 08/21]	**02/2024** [09/23, 10/24]	**08/2028** [06/27, 04/30]	**08/2034** [06/32, 09/37]
Berlin-Brandenburg	**10/2019** [10/19, 10/19]	**09/2020** [09/20, 09/20]	**03/2022** [02/22, 05/22]	**11/2024** [07/24, 04/25]	**06/2028** [10/27, 04/29]
Bremen	**02/2020** [11/19, 07/21]	**10/2022** [09/21, ≥ 2050]	**07/2027** [10/23, ≥ 2050]	**06/2035** [02/27, ≥ 2050]	**12/2045** [06/31, ≥ 2050]
Hamburg	**10/2019** [10/19, 10/19]	**12/2019** [11/19, 08/20]	**04/2022** [01/22, 08/22]	**12/2024** [03/24, 26/05]	**07/2028** [11/26, 05/31]
Hesse	**10/2019** [10/19, 10/19]	**10/2020** [09/20, 10/20]	**06/2024** [09/23, 06/26]	**09/2030** [04/28, 01/37]	**01/2039** [05/34, ≥ 2050]
Mecklenburg Western Pomerania	**08/2022** [05/21, ≥ 2050]	**09/2027** [06/23, ≥ 2050]	**01/2039** [05/27, ≥ 2050]	** ≥ 2050** [02/34, ≥ 2050]	** ≥ 2050** [01/43, ≥ 2050]
Lower Saxony	**08/2020** [06/20, 09/20]	**02/2023** [08/22, 06/24]	**06/2028** [05/26, 10/33]	**07/2037** [09/32, 12/49]	**08/2049** [03/41, ≥ 2050]
North Rhine-Westphalia	**11/2019** [11/19, 12/19]	**10/2020** [10/20, 11/20]	**02/2024** [08/23, 10/24]	**09/2029** [02/28, 10/31]	**01/2037** [02/34, 01/41]
Rhineland Palatinate	**09/2020** [08/20, 11/20]	**05/2023** [09/22, 01/25]	**01/2029** [07/26, 01/36]	**10/2038** [02/33, ≥ 2050]	** ≥ 2050** [11/41, ≥ 2050]
Saarland	**11/2019** [11/19, 12/19]	**11/2022** [03/22, ≥ 2050]	**09/2028** [01/25, ≥ 2050]	**07/2038** [12/29, ≥ 2050]	** ≥ 2050** [06/36, ≥ 2050]
Saxony	**11/2019** [11/19, 01/20]	**11/2021** [09/21, 03/22]	**12/2025** [07/24, 01/30]	**01/2033** [05/29, 09/43]	**06/2042** [10/35, ≥ 2050]
Saxony-Anhalt	**10/2020** [03/20, 04/21]	**11/2022** [04/22, 05/24]	**01/2027** [12/24, 01/33]	**02/2034** [07/29, 02/48]	**07/2043** [06/35, ≥ 2050]
Schleswig–Holstein	**09/2021** [05/21, 03/23]	**09/2025** [09/23, 02/42]	**05/2034** [08/28, ≥ 2050]	**04/2049** [11/36, ≥ 2050]	** ≥ 2050** [08/47, ≥ 2050]
Thuringia	**05/2021** [11/20, 08/22]	**12/2024** [03/23, ≥ 2050]	**02/2033** [03/27, ≥ 2050]	**12/2046** [12/33, ≥ 2050]	** ≥ 2050** [11/42, ≥ 2050]
**Germany**	**11/2019** [11/19, 11/19]	**2021–03** [03/21/03, 04/21]	**02/2024** [11/23, 05/24]	**01/2029** [05/28/05, 29/10]	**2035–08** [05/34, 11/36]

## Discussion

Globally, the WHO estimates that the number of PrEP users increased by 69% from 370,000 in 2018, to about 626,000 PrEP users across 77 countries in 2019 [28]. However, these estimates are inherently uncertain, as data on actual PrEP use is often not available, or needs to be extrapolated between different countries and regions, or, as in the case of Germany, only existed as point estimates [22]. To date, there is no electronic health recording system in Germany to directly monitor the number of PrEP users over time. To overcome this knowledge gap, we developed a mathematical model that allows to calculate past, present and future PrEP use from anonymous antiretroviral prescription data. Our model can distinguish between FTC/TDF prescriptions used for PrEP vs. HIV therapy (and PEP) and accounts for trend changes due to COVID-19 lockdowns. Using our model, we were able to estimate the effect size of COVID-19 lockdowns, the absolute numbers of PrEP users in Germany, as well as regional differences. Additionally, we estimated and forecasted PrEP coverage of people in need of PrEP, for the entire country and within the distinct German federal states. Notably, our approach could be adapted to other countries using anonymous prescription data and hence contribute to improve predictions on global PrEP use as well.

Since the absolute number of PrEP users does not clearly reveal PrEP needs or when PrEP needs are met, we calculated PrEP coverage and predicted when PrEP needs will be met in the future (Fig. 2, Table 3). It is important to note that the calculation of PrEP needs was based on data from the 2017 European-MSM-Internet-Survey (EMIS-2017) on sexual behavior and attitudes towards PrEP, which may have changed since then and will require updating [22]. However, a recent study from the Netherlands that modeled the epidemiological impact and cost-effectiveness of expanding PrEP provision to PrEP-eligible/intending MSM also utilized the Dutch subsample of the EMIS-2017 to define PrEP eligible MSM [29]. The authors estimated that approximately 35% of HIV-negative MSM were PrEP-eligible and the resulting PrEP coverage was 30% in the Netherlands [29]. In the underlying estimation for Germany it was assumed that 1.5% of the adult male population are gay [30, 31]. The distribution of the gay population across federal states in Germany was estimated based on the relative federal state distribution of EMIS-2017 respondents [27]. The estimated PrEP need was 23% in Germany using a total population size estimate of 350,000 adult gay men not diagnosed with HIV living in Germany [22, 27]. In the US, where PrEP was approved already in July 2012, using prescription data from a pharmacy database it was estimated that 365,711 persons were prescribed PrEP in 2021 of whom 337,697 were men and 28.014 women. However, the number of persons with indications for PrEP was estimated at more than 1.2 Million and therefore PrEP coverage was only 30% overall with 34% PrEP coverage in men and 12% in women [32]. Of note, different definitions for PrEP need, and different calculations of the size of the population(s) in need have been used in Germany, the Netherlands, and the US, making direct comparisons of the data problematic.

In Germany, we estimated the coverage of PrEP needs at 33% by Dec. 2021, a similar proportion as in the study from the Netherlands and the US. According to past trends and the projections of our model, coverage of PrEP needs was 14% in 2019, 24% in 2020, and it will be 62% in 2025 and 81% in 2030, if PrEP uptake dynamics remained identical. We also observed clear regional differences in PrEP use with the highest PrEP coverage in the metropolitan federal states Berlin and Hamburg and the lowest coverage in less populated and territorial states (Fig. 2). The regional differences in PrEP uptake very likely reflect different PrEP needs but likely also different structures in HIV care. Our PrEP need estimates for Germany already take regional differences in sexual activity, partner numbers, and condom use into account. On the one hand, more MSM with PrEP needs live in metropolitan areas, and on the other hand, there are more HIV specialty care centers which are the main PrEP providers in Germany due to current regulations. Further, it is important to keep in mind that our data source does actually not indicate where individuals live, but rather where they receive their medicine. For example, cross-state coverage is common for HIV treatment prescriptions [9]. However, recent data from the ‘PrEP evaluation’ (EvE-PrEP) and the ‘PrEP Suveillance’ (PrEP-Surv) projects in Germany revealed gaps in PrEP provision and reaching capacity limits in some regions. In surveys among HIV specialty care centers in PrEP-Surv, 90% of centers indicated gaps in HIV care in rural areas and 76% of centers indicated gaps in HIV care due to a lack of PrEP prescribers in general [24]. Discussions with the PrEP-Surv Community Advisory Board also suggested gaps in PrEP coverage including difficulties in finding a PrEP provider, waiting lists or long distances [20, 33]. Globally, a lack of care structures is believed to be an important barrier to accessing PrEP [34]. Results from Germany point in the same direction and a broader PrEP care structure that also includes general medicine, gynecology, travel medicine, psychiatry and more is highly recommended.

Our data source initially represents FTC/TDF prescriptions within the German SHI system. In order to estimate the total number of PrEP users, we extrapolated 10.5% non-SHI PrEP prescriptions [24, 26]. Moreover, as the number of prescribed tablets is equal to the number of PrEP users only in the case of daily PrEP use, we considered on-demand PrEP use based on detailed and valid results from the PrEP evaluation on the number of pills prescribed as PrEP divided by the number of days on PrEP [25]. The results on PrEP pill coverage were also confirmed in routine data analyses conducted as part of the PrEP evaluation [33]. Nevertheless, the proportion of non-SHI PrEP, as well as on-demand PrEP use remain somewhat uncertain and they could differ by PrEP prescription route (SHI vs. non-SHI) or change over time. We deliberately remained conservative in the lower range compared to previous estimates, especially regarding PrEP on-demand use [22]. Using our method, we estimated that 26,159 individuals used PrEP in Germany at the end of 2021 and we forecasted 49,308 and 64,794 PrEP users in 2025 and 2030 respectively.

However, our long-term predictions are subject to a degree of uncertainty, as we could not take future developments into account, such as the roll-out of novel PrEP-regimen, including long-acting drugs, or major changes to the PrEP provision infrastructure. Our PrEP need calculation is based on data for MSM as the majority of PrEP users in Germany are currently MSM with about 98% [20, 24, 25]. This proportion is in line with data from other countries (Australia, USA, Netherlands). However, this may change in the future and PrEP need in heterosexuals and other populations may increase in the future.

Changes in regulations, prescribing patterns and preferences regarding therapy options could also influence the proportion of FTC/TDF in HIV therapy. The decline in FTC/TDF prescriptions for HIV treatment and PEP before September 2019 for example reflects the increasing use of single tablet regimens and tenofovir alafenamide fumarate (TAF) in HIV treatment in Germany. In 2020 FTC/TAF became a reference price level drug in Germany [35], which could result in co-payment by HIV positive persons and therefore lead to an increase in FTC/TDF in HIV treatment due to re-switch. However, single tablet regimens are exempt from this reference price level regulation and studies show that single tablet regimens are mainly used in HIV treatment [36–38]. Further, weight gain with TAF has been reported [39, 40] which could lead to requests of TDF by HIV positive individuals. However, as mentioned single tablet regimens containing FTC and TDF are preferred and this would not affect our calculations as we only consider single FTC/TDF. Nevertheless, a limitation of our model is the assumption that the trend in FTC/TDF prescriptions for HIV therapy and PEP remains similar after Sept/2019, compared to before that time. Any drastic change in prescription behavior with regard to single FTC/TDF could lead to an under- or overestimation of the number of PrEP users and PrEP coverage. However, the FTC/TDF prescriptions for PrEP appear to far outweigh prescriptions for HIV treatment and therefore the impact of changing treatment prescriptions can be assumed to have comparably minor effects on our predictions.

Among European countries, France was one of the first to introduce PrEP through statutory health insurances. A recent analysis, based on electronic patient record data, indicated that about 42,000 individuals had initiated PrEP by June 2021, with marked effects of COVID-19 on PrEP roll-out [12], similar to our analysis. Without COVID-19 disruptions, the WHO estimated 0.9–1.1 million PrEP users globally by the end of 2020 and 2.4–5.3 million by the end of 2023 [28]. If COVID-19 disruptions resulted in no PrEP user growth in 2020, the projected number of PrEP users in 2023 was 2.1–3.0 million [28]. The CDC recently proposed that the growth in PrEP use, along with increased testing and treatment has played a major role in recent decreases in new HIV infections in the US with an estimated 8% decrease in new HIV infections from 2015 to 2019 after a period of general stability [41]. The impact of COVID-19 related disruptions in HIV prevention services on these trends, however, is not yet known [42].

The impact of COVID-19 on PrEP use in Germany has been described previously in the PrEP evaluation study, showing a profound decrease in PrEP demand, especially in PrEP initiations, an increase in PrEP interruptions and discontinuations, as well as a switching to on-demand PrEP use [26, 43]. In addition, precarious conditions have increased, which also have negative effects on health behavior and prevention efforts [44–46]. The data analyzed here showed a decline in PrEP prescriptions and in the number of PrEP users during COVID-19 lockdowns, with regional differences in the lockdown effects. Overall, a larger effect and decrease in the number of prescriptions and PrEP users was observed in the first lockdown. This is in accordance with other studies that also indicate that the COVID-19 pandemic rather temporarily affected health care seeking and sexual behaviour among certain groups [47, 48]. Notably, in this anonymous data source, no behavioral data and neither persons characteristics such as gender or age are available. Therefore, changes in number of prescriptions during COVID-19 cannot be directly linked with behavior. Furthermore, in our model on-demand use was calculated as stable over time. However, data from studies and surveys in Germany strongly suggest the association between decreased PrEP demand and behavioral changes during and due to COVID-19 lockdowns [26, 49]. Although not directly linkable, we assume that the observed decline in prescriptions and PrEP users primarily reflects changes in PrEP use. As HIV treatment is a vital, lifelong, daily therapy, we assume that HIV treatment was continued throughout the COVID-19 pandemic [50]. The decrease of 300 new HIV infections in 2020 compared to the previous year [9] also does not explain the observed decrease in the number of PrEP users, which was about 10 times higher.

Since HIV PrEP is usually a temporary preventive measure rather than a permanent tool, where individuals engage in PrEP care during periods of heightened HIV risk and discontinue when the risks diminish, the collective of PrEP users is not the same over time. However, as previously described, our data source is not person-specific and therefore it cannot be verified whether these collective of PrEP users is composed of the same individuals. PrEP interruptions or PrEP (re)initiation cannot be directly observed. Nevertheless, this is of secondary importance for the calculation of need coverage, since similar behavioral patterns may apply to the collective of people in need.

Notably, prescription data from February 2022 onwards could be affected by refugees following the Russian invasion of the Ukraine, through increase in treatment or PrEP prescriptions, as refugees from Ukraine are covered by SHI in Germany. However, we only used data until Dec. 2021, consequently our estimations are not affected.

## Conclusions

In summary, our approach presents a comprehensive solution for analyzing and forecasting trends in PrEP use and PrEP coverage from anonymous data, accommodating external influences, thereby contributing to a more informed and effective PrEP strategy. Notably, our approach could be adapted to other countries using anonymous prescription data and hence contributing to improve predictions on global PrEP use.

We saw a diverse picture of PrEP coverage, while in the metropoles of Berlin and Hamburg almost 50% coverage was achieved in 2021, PrEP coverage was only about 10% in other more rural regions. An extension of PrEP care to other medical areas such as general medicine, gynecology, travel medicine, psychiatry and more should definitely be sought. Equally important is the integration of community-based structures, particularly for pre-PrEP counselling, in order to guarantee PrEP care and to relieve existing care structures that are in some areas already working at the capacity limit. Education of public and healthcare professionals about PrEP and key population-specific information on PrEP will be important in order to extend PrEP care and to reach a larger proportion of those who would benefit from PrEP. This would help to ensure greater access to PrEP and progress in PrEP implementation to reach the Sustainable Development Goal of ending the AIDS epidemic by 2030.

Another aspect is ensuring supply even during crises such as the COVID-19 pandemic. We saw a significant effect of the first and second COVID-19 lockdowns on PrEP use. The long-term effects beyond these immediate effects are however speculative, nevertheless it is important to continuously ensure PrEP supply and to work on overcoming negative effects.

### Supplementary Information

Supplementary Material 1.

## Data Availability

All analyses were performed in Python 3.12 using custom codes. Codes and data are available under https://github.com/KleistLab/PrEP_Prescription
