# Supplementary material for: Dynamics of HIV PrEP use and coverage during and after COVID-19 in Germany
Source: BMC Public Health. 2024 Jun 25;24:1691. doi: 10.1186/s12889-024-19198-y (PMC11197325; doi:10.1186/s12889-024-19198-y)

Supplementary Figures and Tables

Supplementary Table S1: Median relative coverage of individuals in need of PrEP [95% CI] over time for each federal German state.

state	2019-12	2020-06	2020-12	2021-06	2021-12	2025-12	2030-12
Baden-Württemberg	**0.063** [0.05, 0.077]	**0.06** [0.043, 0.077]	**0.14** [0.123, 0.155]	**0.165** [0.148, 0.18]	**0.191** [0.17, 0.213]	**0.375** [0.272, 0.463]	**0.546** [0.377, 0.67]
Bavaria	**0.169** [0.16, 0.18]	**0.151** [0.138, 0.163]	**0.22** [0.209, 0.231]	**0.249** [0.238, 0.259]	**0.305** [0.29, 0.319]	**0.624** [0.571, 0.673]	**0.825** [0.771, 0.869]
Berlin-Brandenburg	**0.185** [0.178, 0.192]	**0.169** [0.159, 0.178]	**0.339** [0.331, 0.347]	**0.394** [0.388, 0.4]	**0.468** [0.458, 0.478]	**0.812** [0.787, 0.834]	**0.949** [0.933, 0.96]
Bremen	**0.081** [0.029, 0.131]	**0.07** [0.006, 0.135]	**0.154** [0.091, 0.217]	**0.162** [0.094, 0.226]	**0.195** [0.109, 0.283]	**0.431** [0.002, 0.686]	**0.63** [0.0, 0.89]
Hamburg	**0.251** [0.231, 0.271]	**0.242** [0.217, 0.267]	**0.332** [0.309, 0.352]	**0.391** [0.372, 0.411]	**0.465** [0.437, 0.494]	**0.807** [0.732, 0.864]	**0.946** [0.893, 0.974]
Hesse	**0.204** [0.192, 0.215]	**0.192** [0.176, 0.208]	**0.281** [0.267, 0.294]	**0.306** [0.295, 0.317]	**0.343** [0.327, 0.359]	**0.578** [0.485, 0.648]	**0.757** [0.629, 0.834]
Mecklenburg-Western Pomerania	**0.008** [0.0, 0.04]	**0.034** [0.0, 0.077]	**0.042** [0.0, 0.085]	**0.065** [0.025, 0.102]	**0.082** [0.026, 0.132]	**0.203** [0.0, 0.422]	**0.332** [0.0, 0.654]
Lower Saxony	**0.074** [0.063, 0.086]	**0.086** [0.072, 0.101]	**0.129** [0.117, 0.142]	**0.152** [0.143, 0.162]	**0.184** [0.169, 0.2]	**0.398** [0.299, 0.479]	**0.588** [0.436, 0.697]
North Rhine-Westphalia	**0.13** [0.122, 0.137]	**0.185** [0.175, 0.196]	**0.272** [0.263, 0.281]	**0.307** [0.298, 0.315]	**0.349** [0.338, 0.361]	**0.604** [0.556, 0.653]	**0.787** [0.73, 0.839]
Rhineland Palatinate	**0.06** [0.046, 0.073]	**0.066** [0.049, 0.083]	**0.124** [0.11, 0.138]	**0.144** [0.132, 0.156]	**0.174** [0.155, 0.195]	**0.379** [0.277, 0.469]	**0.564** [0.399, 0.686]
Saarland	**0.124** [0.103, 0.147]	**0.12** [0.094, 0.149]	**0.157** [0.135, 0.182]	**0.174** [0.156, 0.193]	**0.202** [0.173, 0.233]	**0.397** [0.179, 0.562]	**0.576 [**0.183, 0.784]
Saxony	**0.111** [0.097, 0.123]	**0.115** [0.098, 0.133]	**0.187** [0.171, 0.203]	**0.223** [0.21, 0.236]	**0.26** [0.24, 0.28]	**0.501** [0.385, 0.593]	**0.694** [0.523, 0.801]
Saxony-Anhalt	**0.058** [0.036, 0.078]	**0.067** [0.041, 0.095]	**0.113** [0.089, 0.137]	**0.142** [0.119, 0.161]	**0.182** [0.151, 0.216]	**0.447** [0.305, 0.571]	**0.661** [0.451, 0.801]
Schleswig-Holstein	**0.004** [0.0, 0.017]	**0.01** [0.0, 0.028]	**0.05** [0.031, 0.069]	**0.089** [0.071, 0.106]	**0.11** [0.085, 0.135]	**0.261** [0.126, 0.379]	**0.416** [0.167, 0.59]
Thuringia	**0.049** [0.03, 0.067]	**0.037** [0.014, 0.059]	**0.086** [0.061, 0.11]	**0.105** [0.08, 0.125]	**0.127** [0.093, 0.158]	**0.288** [0.108, 0.436]	**0.446** [0.105, 0.66]
**Germany**	**0.14** [0.136, 0.143]	**0.146** [0.141, 0.15]	**0.24** [0.237, 0.245]	**0.278** [0.274, 0.281]	**0.327** [0.322, 0.332]	**0.616** [0.595, 0.638]	**0.81** [0.787, 0.832]

*Supplementary Figure S1: Change in PrEP prescription rates before and during the first COVID lockdown (April 2020). Boxplots show median change in rates and interquartile ranges. Whiskers extend to 1.5 times the interquartile range and dots represent outliers. *p<0.05; **p<0.01.*

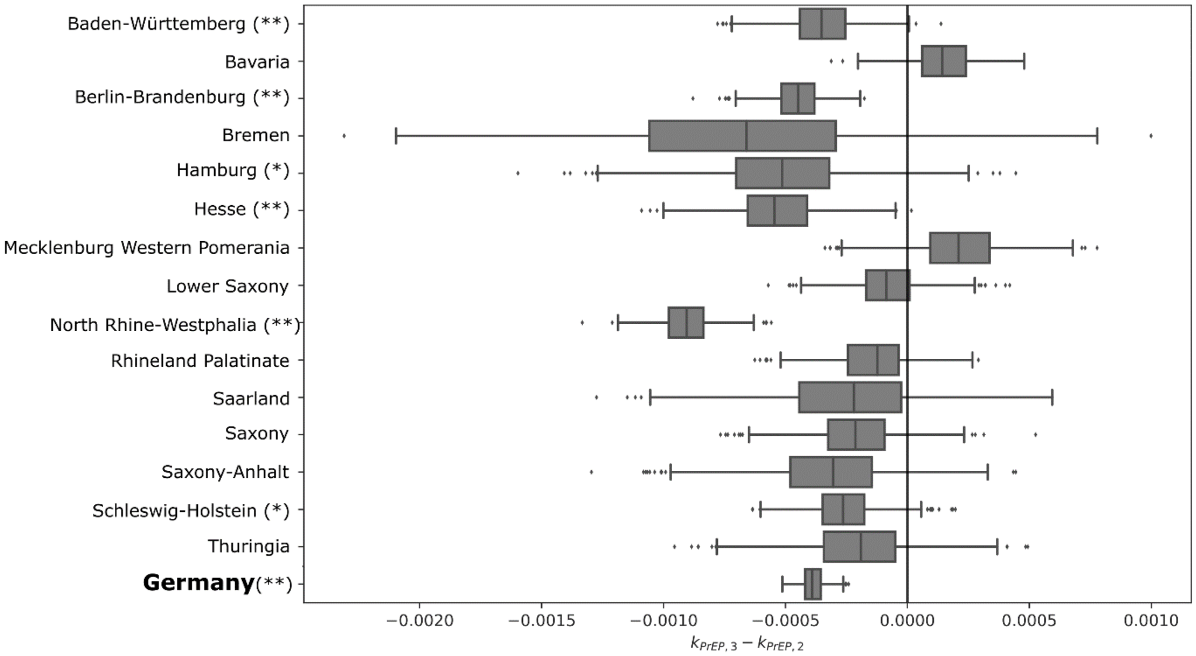

*Supplementary Figure S2: Change in PrEP prescription rates before and during the second COVID lockdown (December 2020). Boxplots show median change in rates and interquartile ranges. Whiskers extend to 1.5 times the interquartile range and dots represent outliers. *p<0.05; **p<0.01.*

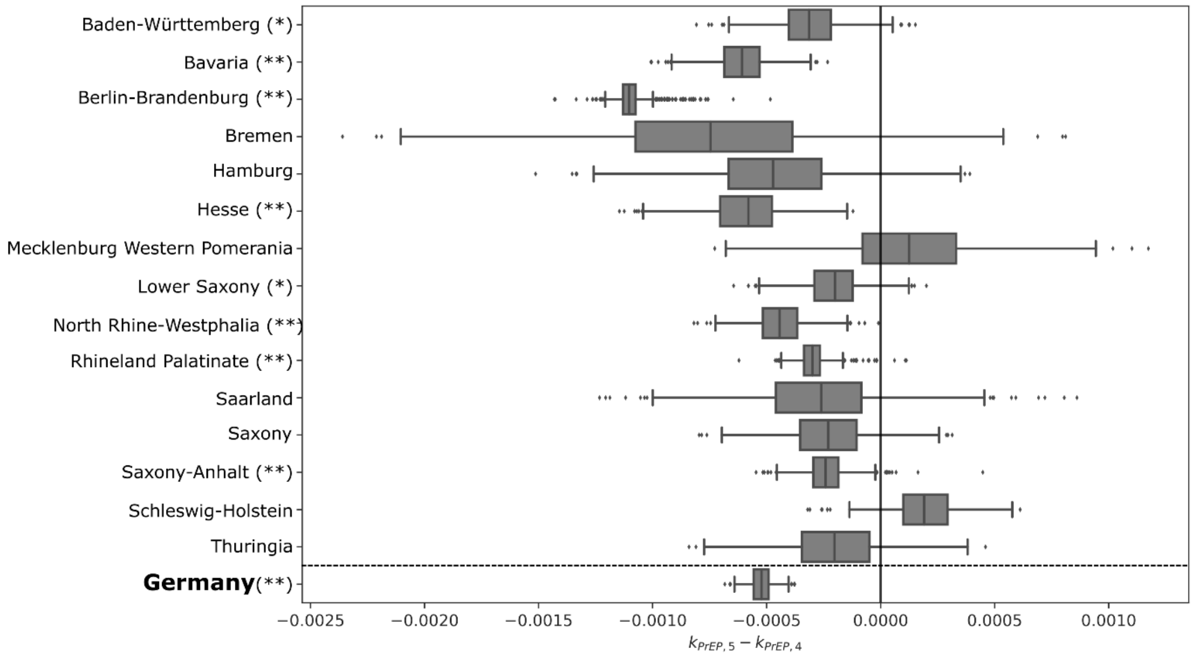

Supplement: Supplementary file 1 — Supplementary Material 1. [file 12889_2024_19198_MOESM1_ESM.pdf]
